# Microsimulation reveals that medically assisted reproduction is unlikely to compensate for cohort fertility decline due to increasing maternal ages

**DOI:** 10.1093/humrep/deag006

**Published:** 2026-02-18

**Authors:** R Granholm, A E P Cantineau, A Hoek, G Stulp

**Affiliations:** Department of Sociology, University of Groningen, Groningen, The Netherlands; Department of Sociology, Inter-University Center for Social Science Theory and Methodology Groningen, Groningen, The Netherlands; Department of Obstetrics and Gynaecology, University Medical Center Groningen, University of Groningen, Groningen, The Netherlands; Department of Obstetrics and Gynaecology, University Medical Center Groningen, University of Groningen, Groningen, The Netherlands; Department of Sociology, University of Groningen, Groningen, The Netherlands; Department of Sociology, Inter-University Center for Social Science Theory and Methodology Groningen, Groningen, The Netherlands

**Keywords:** medically assisted reproduction, completed cohort fertility, contribution, age, counterfactual, microsimulation

## Abstract

**STUDY QUESTION:**

Can medically assisted reproduction (MAR) compensate for completed cohort fertility (CCF) decline within coresidential unions due to increasing maternal ages among Dutch women born during 1974–1984?

**SUMMARY ANSWER:**

MAR is unlikely to compensate for cohort fertility decline within coresidential unions due to continued increase in maternal ages in our sample cohort of Dutch women born during 1974–1984, even under ideal conditions.

**WHAT IS KNOWN ALREADY:**

The pregnancy- and live birth rates for both expectant management and MAR decline at older reproductive ages. Some infertile couples can conceive naturally without undergoing treatment by trying to conceive for a longer period of time, which complicates estimating the contribution of MAR to cohort fertility.

**STUDY DESIGN, SIZE, DURATION:**

We developed a microsimulation model, which includes MAR, that simulates the reproductive life courses of women. We simulate a sample of 1 000 000 women representing the 1974–1984 Dutch birth cohort. Sample sizes for the input parameters varied from hundreds to thousands to hundreds of thousands depending on the parameter and source (surveys, clinical studies, panel data, population registers).

**PARTICIPANTS/MATERIALS, SETTING, METHOD:**

Our Monte Carlo microsimulation model uses probability distributions and parameters based on representative data sources to determine a woman’s transitions through union and reproductive events across her reproductive life course. We assess the contribution of various components of the MAR process to CCF within coresidential unions and estimate the net contributions of MAR to CCF with counterfactual simulations.

**MAIN RESULTS AND THE ROLE OF CHANCE:**

Increases in the maximum female age at MAR treatment, the time to starting IUI treatment, and the number of IUI cycles did not noticeably increase CCF. Out of the hypothetical policy levers that were adjusted, increasing the share of eligible women who took up MAR from 0% to 100% increased CCF linearly by 0.06 children. Increasing the waiting time to ART treatment after infertility diagnosis from 1 to 12 months reduced CCF by 0.01. Increasing the number of reimbursed IVF/ICSI cycles from 0 to 6 increased CCF by 0.05. The increase tapered off after cycle number 2, and levelled off after cycle number 4. Minimum ages at which MAR treatment was started that were between 20 and 30 resulted in near-identical patterns of change in CCF from the adjustment in the potential policy levers, whereas both the CCF levels and rate of increase or decline were reduced at age 35 years, and further at age 40 years. The main influences on the lower CCF levels and slower rate or increase or decline were the reduction in MAR pregnancy rates at advanced reproductive ages, the increasing probability of intrauterine mortality with age, and time spent trying to conceive naturally before starting MAR treatment. The net contribution of MAR (0.043 children per woman, 2.5% to non-MAR CCF) was 33% smaller than the observed MAR birth share of CCF (0.064 children per woman, 3.7% of simulated CCF including MAR). The relative contribution of MAR was strongest at ages 35–39 (6.0%) and at age 40 years and above (8.6%), and weakest at ages 20–24 (0%) and 25–29 years (1.0%). In absolute number of children born, most of the contribution of MAR occurred at ages 30–34 (0.016 children) and ages 35–39 years (0.015). The Monte Carlo variation in the simulation model was measured to be around ±0.001 based on 10 simulations of 1 000 000 women. Parameter uncertainty was accounted for where possible by allowing parameters to vary within nonparametrically bootstrapped values generated from the sample data.

**LIMITATIONS, REASONS FOR CAUTION:**

Our model cannot fully capture the complexity of the fertility and MAR processes with regards to factors like infertility diagnosis, partner characteristics, and health status. Due to missing information, an extensive number of parameters, and limited sample sizes when estimating some of the parameters; the model output deviates slightly from the reference data. We cannot establish causality due to endogeneity in the modelling. We are not entirely confident in the zero contribution of IUI, because clinical data suggest that IUI pregnancy rates are higher than expectant management pregnancy rates, but the quality of that clinical data is low and sparse. The scope of the study is limited to births within coresidential unions, because information on unions and births outside of coresidential union was insufficient for modelling purposes.

**WIDER IMPLICATIONS OF THE FINDINGS:**

While MAR remains an important tool to address infertility, our results suggest that it is unlikely to compensate for population fertility decline attributable to postponement of childbearing to older reproductive ages. Failing to account for the counterfactual case that some couples who undergo MAR may conceive naturally given longer expectant management may lead to overestimation of the contribution MAR can make to population fertility.

**STUDY FUNDING/COMPETING INTEREST(S):**

This work was supported by a VIDI grant (VI.Vidi.201.119) from the Netherlands Organization for Scientific Research to G.S. A.H. reports consulting fees by Ferring Pharmaceutical company The Netherlands, paid to institution UMCG, not related to the presented work.

**TRIAL REGISTRATION NUMBER:**

N/A.

## Introduction

A gap between intended family size and completed cohort fertility (CCF), the number of live births per woman among women born during a specific year-range, has been observed across Europe (Testa, 2014; Beaujouan and Berghammer, 2019). A part of this gap can be attributed to some individuals wanting to have children, but being unable to have a child for various reasons (finding the right partner, economic and employment uncertainty, work-life balance, division of household labour etc), including infertility (‘…failure to establish a clinical pregnancy after 12 months of regular, unprotected sexual intercourse or due to an impairment of a person’s capacity to reproduce either as an individual or with his/her partner.’; Zegers-Hochschild *et al*., 2017). Infertility is not always related to biological age (Zorrilla and Yatsenko, 2013; Sang *et al.*, 2023; Benonisdottir *et al.*, 2024), but couples generally find it more difficult to achieve a live birth at older reproductive ages because their fecundity, their biological ability to produce offspring, declines with age (Sauer, 2015). Medically assisted reproduction (MAR) can effectively address infertility. Expanded access to and subsidisation of treatments, increasing mean ages at birth (Passet-Wittig and Bujard, 2021), and improvements in MAR treatments (Harper *et al.*, 2017) have increased use of ART in Europe. ART includes all infertility treatments where eggs or embryos are manipulated: IVF, ICSI, and FET, but not IUI, which is included in the broader definition of MAR (Jain *et al.*, 2025).

Data on ART births are available for many European countries, ranging from less than 1% in Ireland and Lithuania, to 5–6% in Denmark and Austria, to over 9% in Spain in 2018 (Wyns *et al.*, 2022). In the Netherlands, the context of this study, the ART share of total births tripled from around 1% in 1996 to over 3% in 2021 (own calculations based on data from CBS, 2023a; Stichting LIR, 2024). The increase in ART use has prompted research into the contribution ART can make to population fertility (Tierney, 2022; Lazzari *et al.*, 2023; Burgio *et al.*, 2025; Chanfreau *et al.*, 2025). We add to this literature by performing counterfactual simulations to simulate the influence MAR-related policy levers have on CCF, and investigate whether MAR can compensate for the decline in CCF induced by increasing maternal ages. Moreover, we investigate how much the number of births *only* attributable to MAR, which we call the net contribution of MAR, deviates from the *observed* share of MAR births. We estimate the net contribution by accounting for expectant management (actively monitoring a couple’s natural attempts to conceive without intervening with medical treatment) births as counterfactual competing events with MAR births. In layman’s terms, the counterfactual can be described as follows: ‘What if there was no MAR and all couples who underwent MAR instead had to try conceiving naturally for longer periods of time?’. By estimating the net contribution, we gain further insight into MAR’s potential to compensate for CCF decline due to postponement of childbirth to advanced reproductive ages. The difference between the net MAR contribution and the observed MAR share of CCF also provides information about potential over- or underestimation of the MAR contribution when directly using the observed MAR share of CCF.

Previous studies (Leridon, 2004; Habbema *et al.*, 2009, 2015; Leridon and Shapiro, 2017; van Eekelen *et al.*, 2020) have analysed the association between MAR and childbirth in the Netherlands and France using macro- and microsimulation, but our approach makes some new contributions. Our model is more detailed than previous models with regards to unions, the reproductive process, education, contraception, and MAR. For policy implications this is important, because MAR treatments are part of a complex set of decisions made over the reproductive life course. For instance, women with a tertiary level of education who marry are more likely to make use of MAR than unmarried women with a primary education, because the former are more likely to postpone their fertility to older reproductive ages and remain in a stable relationship (Goisis *et al.*, 2020, 2022). We also estimated female age-specific pregnancy rates for IUI (includes both donated and own (heterologous) sperm, as well as both with and without ovarian stimulation), IVF, ICSI, and FET, simulating how the MAR process works in practice as accurately as possible to measure the net contribution of MAR. Moreover, we model the female age-specific probability of a multiple birth occurring for both natural and MAR pregnancies. While the probability of having a multiple birth as a result of MAR treatment has declined over the past two decades due to a shift from multiple embryo- to single embryo transfers (Wyns *et al.*, 2022), it was still considerably higher than for natural conceptions in our sample cohort.

We simulate the CCF within coresidential unions of Dutch women born during 1974–1984, including parameters on education, fertility intentions, partnering-, and contraceptive behaviour, as well as physiological constraints on fecundity. We perform various counterfactual simulations by adjusting our model parameters to measure how much each component of the MAR process can contribute to CCF in our sample cohort as a whole, and at different ages. The counterfactuals focus on specific policy levers such as the upper age limit on MAR treatment reimbursement, the number of reimbursed treatment cycles, waiting times before and between MAR cycles, and the share of women eligible for MAR who take up treatment. We also estimate the net contribution of MAR to CCF. Based on these simulations, as well as simulating an ‘ideal’ MAR scenario, we assess whether MAR can compensate for CCF decline due to increasing maternal ages.

## Materials and methods

### Microsimulation model

Our simulation model is an empirically calibrated Monte Carlo microsimulation. It is an extension of a model developed for a previous study (Granholm *et al.*, 2025), which in turn builds on a model developed by Henri Leridon (Leridon and Shapiro, 2017). The model runs for individual women, and we run the model 1 000 000 times (resulting in a Monte Carlo variation of [−0.0011, +0.0007] from the mean CCF with 10 model runs) to simulate a synthetic cohort. The simulation model iterates monthly, the length of the average menstrual cycle (Bull *et al.*, 2019). The model is split into two parts: the union formation simulation, followed by the reproductive simulation. The union trajectories produced by the union simulation determine whether and when the woman (couple) tries to conceive a child in the reproduction simulation. Each woman enters the simulations at age 15 years and the simulation stops at age 55 years. Supplementary Figures S1, S2, S3, and S4 depict how the different parts of the simulation work. To construct the model, we used the software R and the RStudio integrated development environment.

### Data

We used data from a wide range of sources to construct model parameters and distributions (see Supplementary Table S1). Most of the behavioural parameters are estimated from Generations and Gender Survey (GGS) Wave 1 (2003) (Gauthier *et al.*, 2018; Generations and Gender Programme, 2019) and the first 15 waves (2008–2022) of the Family and Household questionnaire as well as background variables in the Longitudinal Internet studies for the Social Sciences (LISS) panel administered by Centerdata (Tilburg University, The Netherlands). We restricted our sample to Dutch women born during 1974–1984 with a reported level of education. See Granholm *et al.* (2025) for a more detailed description of the behavioural data and parameters that are not related to MAR (Granholm *et al.*, 2025).

### MAR pregnancy rates

For MAR per-cycle pregnancy rates, we used the female age-specific IVF pregnancy rates from Habbema *et al.* (2015). We transformed the data from years to months and interpolated using a cubic spline to get a monthly distribution. We then calculated mean female age-specific pregnancy rates across MAR treatment types for the year 2013 using data from Stichting LIR (2024), except for IUI, where we used a Danish estimate from 2013 (Sundhedsdatastyrelsen, 2024). To get monthly female age-specific pregnancy rates for all four MAR treatment types, we multiplied the female age-specific IVF pregnancy rates with the ratio between the mean IUI/ICSI/FET and the IVF pregnancy rate, assuming that the shape of the female age-specific probability distribution was the same across treatment types. We conducted sensitivity analyses where we adjusted both the FET and IUI curves due to their limited influence on CCF in our analysis. The sensitivity analyses can be found in the GitHub repository: https://github.com/rofa-g/lifert_mar.

To reproduce MAR use in our chosen cohort, we needed reference estimates for the IUI and ART shares of total births in the Netherlands. The ART shares were calculated directly from Dutch ART (Stichting LIR, 2024) and birth registers (CBS, 2023a), by averaging over the years 2003, 2010, and 2020. For IUI births, we used Danish data (mean of the years 2013 and 2018) (DST, 2024; Sundhedsdatastyrelsen, 2024) as a proxy because there was no reporting in the Netherlands. To estimate the share of IUI births in the Netherlands, we calculated the ratio of ART births in 2019 in Denmark and the Netherlands and multiplied this ratio with the Danish IUI share estimate. These calculations resulted in a MAR share of total births of 3.7% in our simulated cohort: 1.3%-points from IUI births, 2.4%-points from ART births. We used nonparametric bootstrapping to generate 10 000 probabilities of MAR uptake from which each individual woman was assigned a random value. The bootstrap was based on a mean of 73% and a sample size of 825 from the 27% share of subfertile couples who participated in a follow-up of a prospective cohort study in the Netherlands (2002–2004), who neither started MAR treatment, nor became pregnant within 12 months of completing fertility workup (van der Steeg *et al.*, 2007).

We set our simulation to produce the reference shares of IUI and ART births by adjusting the fecundability (monthly probability of conceiving when not using any contraceptive methods) threshold (<0.0077) for transition to ART and the fecundability criteria for entry into MAR (<58% probability of pregnancy within 12 months of infertility diagnosis) for women below age 38 years. The MAR entry criteria was based on the Hunault model used in Dutch fertility workups to predict the probability of natural conception. The Hunault criterion for starting fertility treatment in the Netherlands is a predicted probability of pregnancy below 30% in the 12 months following fertility workup. However, ‘overtreatment’ (starting MAR treatment despite having a probability of pregnancy above 30% in the 12 months following fertility workup) was found to occur in a third of couples with unexplained infertility who were eligible for 6 months of expectant management (Kersten *et al.*, 2015, 2016). Directly using the 30% Hunault criteria in our simulation model resulted in a lower IUI share (0.9%) and ART share (2.2%) of CCF than the estimated observed IUI and ART shares of 1.3% and 2.4%. For information about the Hunault model, see Hunault *et al.* (2004, 2005).

### Multiple births

Information on the female age-specific probability of having a multiple birth was accessed from a report by Statistics Netherlands (CBS, 2016). This data has the shortcoming of including all births, both natural and MAR births. We downward adjusted the natural multiple birth rates derived from the report to account for this. We used the adjusted mean values of each age group over the years 1995, 2005, 2015; transformed the age data from years to months, and interpolated data points using a cubic spline to get a monthly probability distribution. To get the probabilities of multiple birth for each MAR treatment type, we first calculated mean probabilities of multiple birth across all women undergoing each type of treatment. In the case of IVF, ICSI, and FET, we used Dutch ART register data for the years 2003, 2010, and 2020 (Stichting LIR, 2024). For IUI, we used Danish data for the years 2007 and 2018 from European Society of Human Reproduction and Embryology reports (de Mouzon *et al.*, 2012; Wyns *et al.*, 2022). We then divided these means by the probability of natural multiple birth to get ratios, which we multiplied with the female age-specific probability distribution of having a multiple birth. This resulted in monthly age-specific probability distributions of multiple birth for each MAR treatment type, assuming that the shape of the distribution was the same for MAR and natural births. We calculated the ratio of twin to triplet births from the same data sets.

### Model validation

Roughly 10% of births occurred outside of coresidential unions in our sample cohort (births outside of coresidential union made up 7.1% of total births in 1996, 10.4% in 2011, and 11.3% in 2022. Given that mean age at first birth was 29 years for our sample cohort, most births occurred during the 2000s and 2010s (CBS, 2023b)). Conceptions in our model only occur while the woman is in a coresidential union, but some unions dissolve during pregnancy, resulting in 2.3% of births occurring outside of coresidential unions in the baseline model run. As we lacked information on births outside of coresidential unions and the transition probabilities between non-coresidential and coresidential union events, we were unable to model the remaining 7.7% of births outside of coresidential unions (see brief discussion on this in the table notes of Supplementary Table S2 and Granholm *et al.*, 2025). This omission should not have induced substantial bias in our estimate of the MAR contribution to coresidential CCF because Dutch fertility clinics have been reluctant to provide MAR treatment to single women (NVOG, 2016), and because less than 10% of Belgian (no Dutch data available) women who gave birth as a result of ART during 2010–2019 were single (Schnor, 2022). The other simulation outputs were close to the reference estimates, with some caveats briefly discussed in Supplementary Tables S2 and S3.

Our model produced higher MAR treatment use among women with a tertiary level of education (Supplementary Tables S4 and S5), which is in line with the empirical literature. The educational gradient in our model, which is only based on later partnering and first conceptions among secondary and tertiary educated women, is weaker than what we would expect from European data (60–65% of all MAR children being born to tertiary educated mothers). This could suggest that the educational difference in MAR use is not only due to postponement of childbearing to older reproductive ages (Goisis *et al.*, 2020, 2022).

## Results

### Counterfactual simulations

To investigate the contribution of MAR to CCF, we ran a number of counterfactual simulations where we adjusted single MAR-related parameters in the model, while keeping all other parameters constant. We chose the following parameters that could be linked to public policy:

- The share of women (or their partner) with an infertility diagnosis who choose to undergo MAR
- The maximum female age at MAR treatment
- The minimum waiting time from infertility diagnosis to the first MAR treatment cycle, and between consecutive treatment cycles
- The maximum number of reimbursed cycles (six cycles of IUI followed by three cycles of IVF/ICSI with FET). The number of reimbursed treatment cycles reset to their maximum when a MAR pregnancy results in a live birth or intrauterine mortality (foetal death at any point during pregnancy, including both miscarriage and stillbirth). FET is not a part of the counterfactual analysis because it is modelled differently than IUI and IVF/ICSI (see Supplementary Fig. S4 and GitHub repository for details), and did not noticeably contribute to CCF.

and adjusted their levels to estimate their contribution to coresidential CCF. Moreover, we simulated female age-group differences in MAR contributions to CCF to evaluate how female age influences the MAR process with the following parameter adjustments:

- The share of women (or their partner) with an infertility diagnosis who choose to undergo MAR
- The minimum time trying to conceive naturally until infertility diagnosis
- The minimum waiting time from infertility diagnosis to the first IVF/ICSI treatment cycle, and between consecutive IVF/ICSI treatment cycles
- The maximum number of reimbursed IVF/ICSI cycles

We also estimated the net contribution of MAR, which accounts for the counterfactual case that some couples who undergo MAR may have conceived naturally given longer expectant management in the absence of MAR. This was done by comparing a simulation where MAR was unavailable with the baseline simulation.

### Counterfactual simulations with potential policy levers

**Figure 1.**
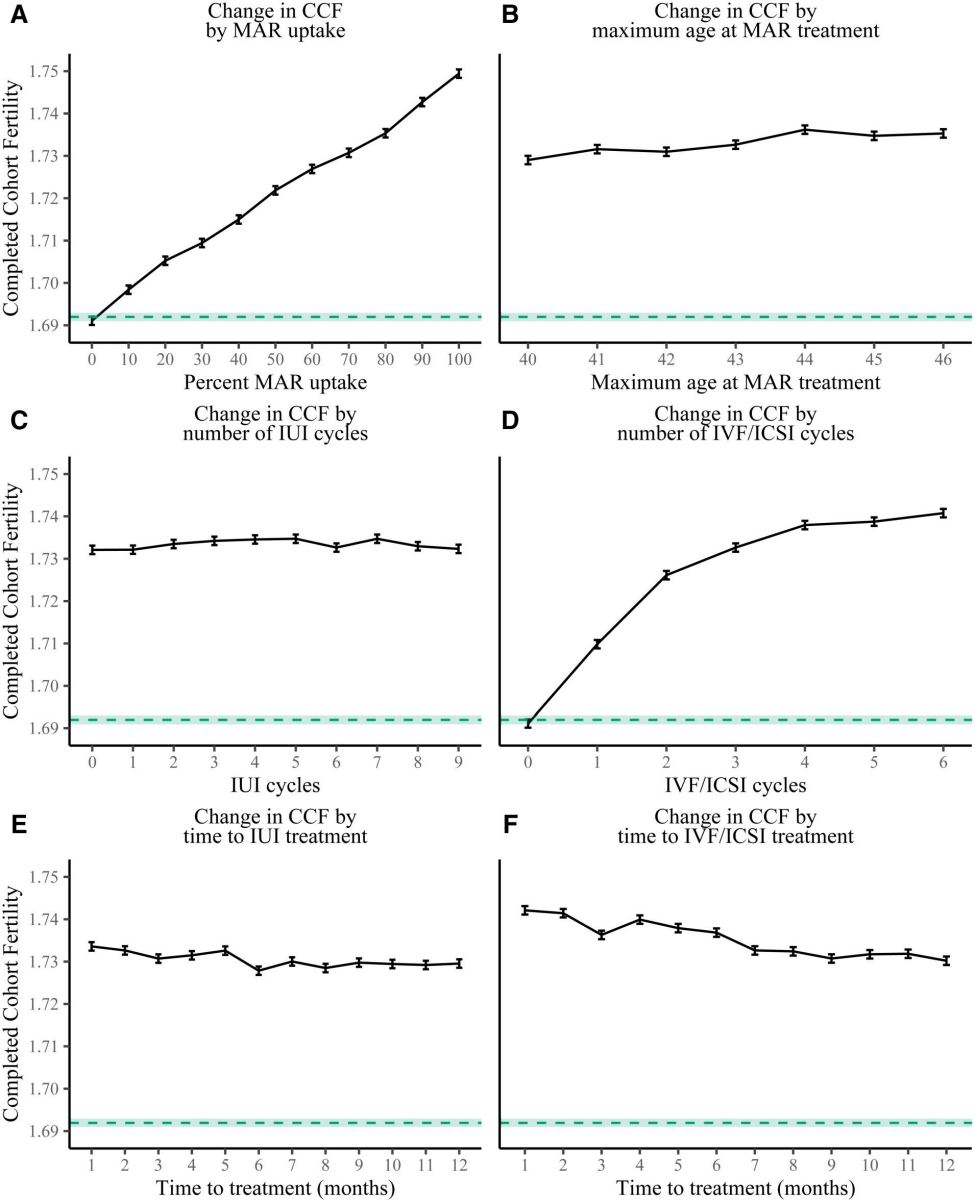
**Contributions of various MAR-related parameter adjustments (counterfactuals) to CCF**. The size of the error bars is ±0.001 based on the variation in cohort fertility from 10 runs of the baseline model. The horizontal green dashed area indicates the case of no MAR treatments occurring in the cohort. Time to treatment here refers to the time until the fertilisation is performed, either for the first or *n*th time. MAR, medically assisted reproduction; CCF, completed cohort fertility.

The share of eligible women who took up MAR treatment made the greatest contribution to CCF (Fig. 1, plot A), and the association was near-linear. Even in the case of 100% MAR uptake, the absolute contribution of MAR to CCF was small at just under 0.06. Adjusting the maximum female age at treatment between ages 40 and 46 years (Fig. 1, plot B) did not noticeably change the contribution of MAR to CCF. This was expected given how few births there are, how low MAR pregnancy rates are, and how high the probability of intrauterine mortality is at ages 40 years and above.

We found that the time between infertility diagnosis and first IUI treatment or between consecutive IUI treatments barely contributed to CCF (Fig. 1, plot E). The number of IUI cycles also had no visible influence on CCF (Fig. 1, plot C). This is may be due to the lenient criteria of starting MAR treatment in our model (<58% probability of conceiving a child in the 12 months following fertility workup vs <30% in the Hunault criteria) for women up to age 38 years, resulting in many women starting IUI treatment with fecundability levels higher or similar to the IUI pregnancy rate. There is also uncertainty in the IUI pregnancy rate relative to expectant management and other treatment types because the existing data on differences in pregnancy rates by MAR treatment type from randomised controlled trials is considered to be of low quality (Wang *et al.*, 2019). However, even when increasing the mean IUI pregnancy rate by more than a third as a sensitivity check, CCF only increase by 0.002. Increasing the time to IVF/ICSI treatment from 1 to 12 months reduced CCF by around 0.02 (Fig. 1, plot F), and increasing the number of IVF/ICSI cycles from 1 to 6 increased CCF by around 0.05 (Fig. 1, plot D). The decline in CCF due to waiting additional months to get treated was almost linear, whereas the increase due to additional IVF/ICSI cycles levelled off after the fourth cycle. The slower increase in CCF from cycle 2 onwards likely happens because the number of reimbursed MAR treatments reset at live births and intrauterine mortality (in case either event occurs, the woman gets another six IUI cycles and/or three IVF/ICSI cycles reimbursed regardless of how many cycles she had gotten reimbursed previously). Each woman also has a limited time window to undergo IVF/ICSI cycles due to spacing, age-related decline in fecundability, and increase in the probability of intrauterine mortality, as well as the probability of experiencing a union dissolution while undergoing treatment.

**Figure 2.**
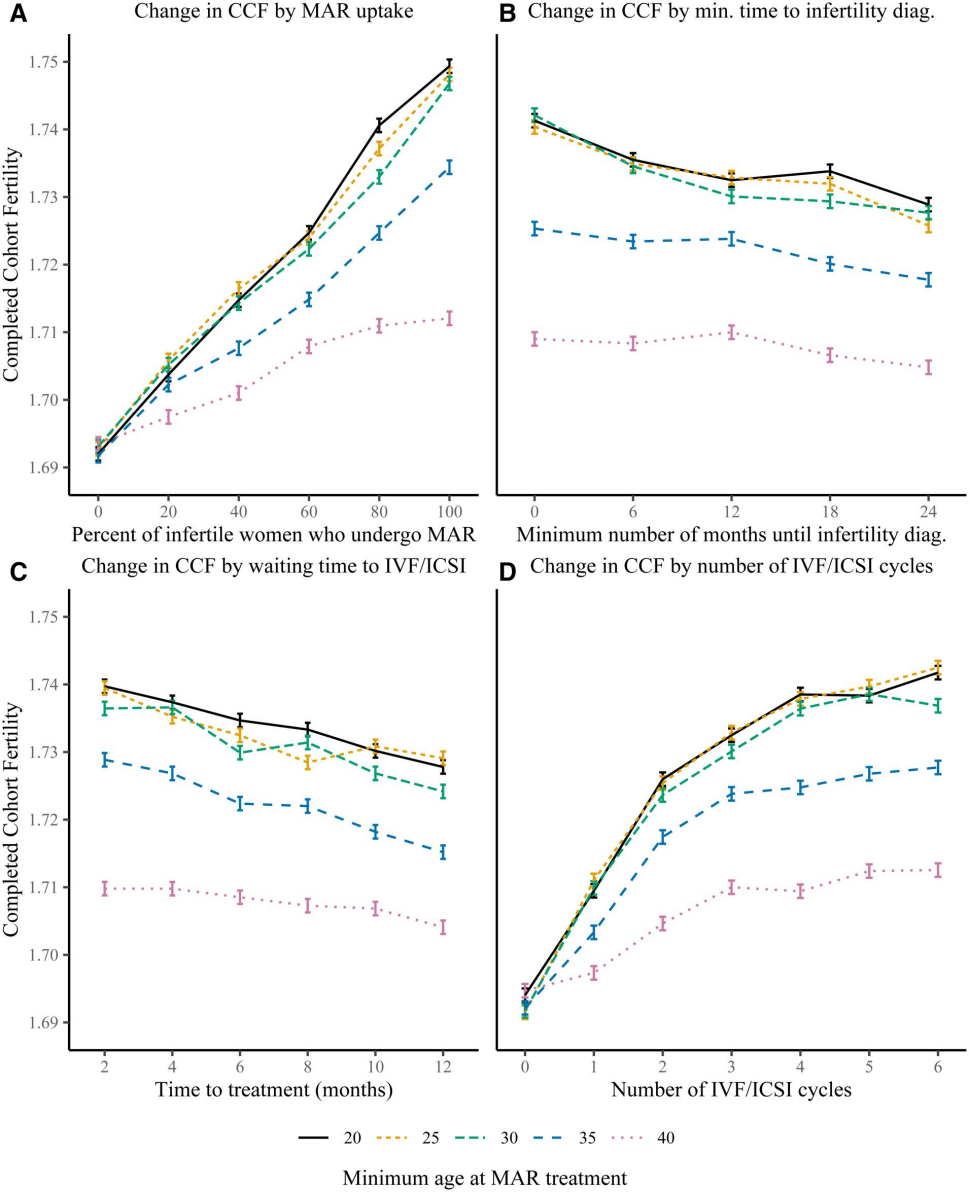
**Contributions of various MAR-related parameter adjustments (counterfactuals) to CCF by minimum female age at MAR treatment**. The size of the error bars is ±0.001 based on the variation in cohort fertility from 10 runs of the baseline model. The minimum waiting time until infertility diagnosis is 12 months in our simulation model. This is the minimum age because women up to age 38 years must have a cumulative probability of conception that is less than 58% over the 12 months following the 12th month of trying to conceive unsuccessfully to start MAR treatment. This check is based on the Hunault criteria. Time to treatment here refers to the time until the fertilisation is performed, either for the first or *n*th time. MAR, medically assisted reproduction; CCF, completed cohort fertility.

When comparing the share of women who underwent MAR after infertility diagnosis by minimum female age at MAR (Fig. 2, plot A), the increase in CCF was strongest, linear, and very similar at ages 20–30 years, and declined substantially by ages 35 and 40 years, while also tapering off slightly in the oldest minimum age at MAR treatment. The total increase from 0% to 100% MAR uptake was around 0.06 at the three youngest ages, 0.04 at age 35 years, and 0.03 at age 40 years. The benefit of earlier infertility diagnosis was similar across all ages (Fig. 2, plot B), with CCF declining by around 0.01 when the minimum time to infertility diagnosis was increased from 0 to 24 months. The fact that the line representing age 35 years was around 0.02 points and age 40 years around 0.03 points lower than the lines representing ages 20–30 years indicates that MAR had a stronger influence on CCF at younger ages. These results are mirrored in the waiting times to first IVF/ICSI treatment and between consecutive IVF/ICSI treatments (Fig. 2, plot C), with slightly smaller differences in the levels of the lines (0.01 for age 35 years, 0.02–0.03 for age 40 years).

There is a slight difference between the two waiting times (Fig. 2, plots B and C) because infertility diagnosis only occurs once, whereas there are multiple IVF/ICSI cycles and therefore multiple waiting times to or between treatment. The three youngest minimum ages at MAR treatment show similar levelling-off in the CCF increase by the fourth IVF/ICSI cycle (Fig. 2, plot D) as in the cohort as a whole (Fig. 2, plot D), increasing CCF by 0.02, 0.02, 0.01, and 0.01 at cycles 1–4. For ages 35 and 40 years, the levelling-off already starts from cycle 3 with CCF increasing by 0.01, 0–0.02, and 0.01 at cycles 1–3. The earlier levelling-off and diminishing increase in CCF per IVF/ICSI cycle at higher reproductive ages again relates to the fact that MAR pregnancy rates decline with age: fewer MAR births would be conceived and the added benefit of additional IVF/ICSI cycles would decline if all women in the cohort started MAR at older reproductive ages. The probability of experiencing an intrauterine mortality would also increase. Moreover, the age differences are influenced by the time spent trying to conceive naturally: the average woman will be more likely to have achieved her desired number of children before making use of MAR, the older she is when she is eligible for her first MAR treatment.

To summarise what our results illustrate about how postponement of reproduction influences CCF in the presence of MAR in a simple manner, we can increase the female age at first cohabitation in our model, which corresponds to an increase in the age at first attempt to conceive, and consequently results in a similar increase in the mean female age at first birth. We then simulate these increases in the age at first cohabitation in an ‘ideal’ MAR setting, where every woman with an infertility diagnosis who has not yet achieved her intended family size undergoes treatment (100% MAR uptake), and there is no upper age limit on MAR treatment (the maximum age in the baseline model is 43 years). We increased the mean female age at first cohabitation by 1, 3, and 5 years (mean age at first birth from 28.9 to 29.8, 31.6, and 33.4, respectively), which reduced CCF by 0.02, 0.08, and 0.16, respectively (MAR share of CCF changed from 5.5% to 5.9%, 7.0%, and 8.0%, respectively). This shows two things. First, the negative influence postponement of reproduction has on CCF strengthens at older reproductive ages. Second, MAR cannot compensate for this increasing loss of CCF, as its ability to increase CCF weakens at older average female ages at treatment.

### Net contribution of MAR

The net contribution of MAR to CCF for the entire cohort was 0.043 children per woman (2.5% increase to the non-MAR CCF of 1.69), 33% smaller than the observed MAR births of 0.064 children per woman (3.7% reference observed MAR share of CCF) (Table 1). The difference between the contributions is the counterfactual that some MAR births would have occurred naturally in the absence of MAR if there had been longer expectant management (based on our model inputs and assumptions). MAR made the greatest relative contribution to CCF at ages 40 years and above (8.6% of all births) and the smallest contribution at ages 20–24 years (0% of all births). But in absolute terms, 0.031 out of the 0.043 CCF contribution (72%) occurred at ages 30–39 years. These results suggest that caution is advised when directly using observed MAR birth shares to calculate the contribution of MAR to CCF, and also strengthen our conclusion that MAR is unlikely to compensate for CCF decline due to continued increase in maternal ages.

**Table 1.** Absolute and relative net contributions of medically assisted reproduction in different age groups.

Indicator	CCF	CCF ages 20–24	CCF ages 25–29	CCF ages 30–34	CCF ages 35–39	CCF ages 40+
Without MAR	1.692	0.172	0.656	0.569	0.236	0.057
With MAR	1.735	0.172	0.662	0.585	0.251	0.063
Absolute difference/contribution	0.043	0.000	0.007	0.016	0.015	0.005
Relative difference/contribution (%)	2.470	0.005	0.998	2.731	5.960	8.564

MAR, medically assisted reproduction; CCF, completed cohort fertility. ‘Without MAR’ refers to a baseline simulation run without MAR use, ‘With MAR’ refers to a baseline simulation run that includes MAR use.

## Discussion

The demand for MAR is expected to increase with continued postponement of childbearing to older reproductive ages, but the mean female age at first birth has now exceeded 30 in countries like the Netherlands. From female ages above 35 years, not only natural pregnancy rates but also MAR pregnancy rates begin to decline sharply, as we have shown in our analysis. ART can still be effective for women up to around age 40 years, but the ART pregnancy rate has been found to be only 10% higher than the expectant management pregnancy rate at ages above 40 years (van Eekelen *et al.*, 2019). Pregnancy rates of ART treatments have remained stable in the Netherlands since the mid-2000s (own calculations based on data from (Stichting LIR, 2024)), and there is limited or no evidence that recent technological innovation in ART improve treatment outcomes (HFEA, 2023, 2024). Furthermore, evidence from Israel suggests that free and accessible IVF (up to age 45 years) may have the unintended consequence of increasing women’s ages at marriage and childbirth, as it extends their perceived fertility window (Gershoni and Low, 2021). In light of this, we found that increasing the current number of reimbursed MAR cycles (six cycles IUI, three cycles IVF/ICSI) in the Netherlands would not noticeably increase population fertility. Even under ideal circumstances with full utilisation of MAR and no upper age limit on reimbursement, MAR could not compensate for CCF decline due to postponement of childbirth to older reproductive ages in our sample cohort. Assuming that the mean female age at first birth will continue to increase, and based on the weakening compensating influence of MAR on CCF at older reproductive ages we found in our analysis, MAR cannot be expected to compensate for the resulting decline in CCF in the Netherlands.

Each couple has a unique and variable fecundity, which is influenced not only by their age but also their health (Ribeiro *et al.*, 2022) and genetics (Zorrilla and Yatsenko, 2013; Sang *et al.*, 2023; Benonisdottir *et al.*, 2024). From a societal standpoint, it could however be useful for young couples and individuals to have a basic understanding about MAR treatments and their limitations when planning their reproductive lives (Hashiloni-Dolev *et al.*, 2011; Daniluk and Koert, 2013; Fauser *et al.*, 2019; Martins *et al.*, 2024). Neither overtreatment with MAR, nor postponement of fertility treatment until pregnancy rates become very low are desirable outcomes for couples or society. Overtreatment poses avoidable health risks to the mother and the foetus (Davies *et al.*, 2017; Kawwass and Badell, 2018; Chih *et al.*, 2021), and takes up (public) resources (van Eekelen *et al.*, 2020; Martins and Connolly, 2022; Feng *et al.*, 2024), possibly limiting access to treatment and increasing waiting times. As our analysis has shown, increased waiting times to MAR treatment after infertility diagnosis results in fewer successful treatments and reductions in CCF. Postponing reproduction and MAR treatment too much can instead result in failure of treatment and involuntary childlessness, which has mental health implications (Tosi and Goisis, 2021; Goisis *et al.*, 2023; Zaks *et al.*, 2023) and contributes to population fertility decline and -aging.

Despite 3.7% (0.064 children per woman) of all births in our simulated sample cohort being MAR births, our counterfactual analysis suggests that the net contribution of MAR to CCF was 33% smaller at 2.5% (0.043 children per woman). Ignoring this counterfactual can inflate contributions MAR make to completed fertility, as suggested by for instance Habbema *et al.* (2009). Comparing these results to other studies analysing the association between MAR and completed fertility, Burgio *et al.* (2025) estimated that the contribution of MAR to total fertility rates (TFR) increased from 2.1% to 3.6% between 2013 and 2022 in Italy. Lazzari *et al.* (2023) projected that the contribution of ART to CCF would almost triple between the 1968 and 1986 Australian birth cohorts from 2.1% to 5.7%, and Tierney (2022) estimated that the contribution of ART to TFR in the USA would double from 1.3% to 2.6% between 2020 and 2040. All three studies directly compared observed MAR births to total births. The two latter studies assumed a continuation in past ART trends. Had these studies adjusted for natural births as possible competing events with ART births as we do in our study, their estimated increases in population fertility would likely have been more modest. Sobotka *et al.* (2008) and Chanfreau *et al.* (2025), studying Danish (1960–1978 cohorts) and Norwegian (1949–1974 cohorts) births, adjusted their estimates to account for natural births replacing ART births, or occurring after ART treatment, by fixed amounts based on data and literature. Both also controlled for multiple birth differences between ART and natural births, and the latter also adjusted their estimates for overtreatment. Their adjusted ART contributions to CCF declined by 15–50% and 35–52%, respectively (Sobotka *et al.*, 2008; Chanfreau *et al.*, 2025). These are similar to the 33% reduction in CCF we observe in our net contribution of MAR despite the differences in methods and contexts.

We make a number of contributions with our study, but it also has some limitations. Despite finding similar results on the net contribution of MAR in other studies, we cannot claim that our results are generalisable to the wider European context. This is because MAR use and availability, MAR pregnancy rates, MAR reimbursement policies, and population age structures vary considerably between countries. The conclusions we can draw are therefore limited to our sample and context. Our modelling approach is not causal due to endogeneity issues. For instance, our model only allows union events to influence childbirth, but not the reverse. We therefore cannot model how having young children could influence the probability of re-partnering. Another endogeneity problem is that we do not include all potential covariates (e.g. employment, income, household division of labour, male partner characteristics), some of which either mediate or confound relationships between our parameters of interest and fertility outcomes.

Information on partnering and childbearing intentions and realisations outside of coresidential unions were too limited for us to incorporate them into our model. With the information we have about births outside of coresidential unions in the Netherlands, we however show that our simulation results are close to what we can expect fertility within coresidential unions to have been in our sample cohort. We also did not have data to distinguish between voluntary termination of an unwanted pregnancy and termination due to health risks to the mother or foetus in medically induced abortions. We modelled all medically induced abortions to be voluntary terminations of unwanted pregnancies, assuming that only a small share of them were due to health risks.

While we are able to model uncertainty in several of the key parameters (timing of union events, fecundability, MAR uptake), for other parameters, this was not possible due to not having access to the original data that was used to produce the parameter estimates or due to how the simulation model is constructed. In some cases, this was less problematic, for instance, where we rely on estimates that were derived directly from population or MAR registers. In other cases where the estimates were based on surveys—as was the case for intrauterine mortality, the age at natural sterility, and unintended pregnancies—modelling the parameter uncertainty would likely have altered our results somewhat. There is also uncertainty in exactly how many infertile couples would conceive naturally without MAR treatment, due to lack of randomised controlled trials comparing expectant management and MAR, and the fact that around 30% of infertile couples have unexplained infertility (Romualdi *et al.*, 2023). This adds uncertainty to our estimated net contribution of MAR to CCF. We also do not explicitly model treatment drop-out (which is somewhere around 15% according to Bensdorp *et al.*, 2016; Leijdekkers *et al.*, 2018), or the potential negative effect of failed MAR treatment on union stability, but including both of these would likely have made our estimated net contribution of MAR even lower.

Furthermore, data were less reliable for parameters such as the IUI pregnancy rate and the difference in treatment success probabilities between different MAR treatment types (Wang *et al.*, 2019), which in our case may have led to an underestimation of the contribution made by IUI. On the other hand, our threshold for entry into MAR (starting with IUI) was below a 58% probability of conceiving the following 12 months. Clinical trials in the Netherlands suggests that in the case of a 30–40% probability of conceiving (moderate prognosis), IUI with ovarian stimulation did not produce markedly higher pregnancy rates than expectant management (Steures *et al.*, 2006), but for couples with a probability below 30% (poor prognosis) IUI with ovarian stimulation did produce higher pregnancy rates (Wessel *et al.*, 2022). We also lacked data on male age and male-factor heterogeneity in relation to MAR pregnancy rates.

At the end of the day, it is important to keep in mind that reality rarely follows an ideal pattern. Couples may not voluntarily postpone their fertility to older reproductive ages. The postponement is a result of decisions (career, preference, commitment) and circumstances (finding a partner, economy, labour market, health) both within and outside a couple’s control. MAR treatments, especially the more invasive IVF/ICSI treatments, are physically and mentally draining processes for both partners involved. Being unable to achieve a live birth through multiple treatment cycles, especially if expectations are high, can be devastating (Brandes *et al.*, 2009; Ying *et al.*, 2016; Goisis *et al.*, 2023). It is therefore important that we strive to address the societal changes that underlie postponement of childbearing and the increasing demand for MAR, and maintain a balanced view of MAR’s potential to address infertility at the societal level.

## Conclusion

We show that even under ideal circumstances with full utilisation of MAR and no upper age limit on reimbursement, MAR cannot be expected to compensate for CCF decline attributable to postponement of childbearing to advanced reproductive ages in the Netherlands. We also found that the net contribution of MAR was 33% smaller than the observed MAR share of CCF within coresidential unions in the Dutch cohort we studied. While our results are non-causal due to endogeneity issues, they do indicate that one should be cautious when making assumptions about, and directly using observed MAR birth shares to predict, MAR’s potential to increase population fertility.

## Supplementary Material

deag006_Supplementary_Figure_S1

deag006_Supplementary_Figure_S2

deag006_Supplementary_Figure_S3

deag006_Supplementary_Figure_S4

deag006_Supplementary_Table_S1

deag006_Supplementary_Table_S2

deag006_Supplementary_Table_S3

deag006_Supplementary_Table_S4

deag006_Supplementary_Table_S5

## Data Availability

The data underlying this article are available in the article and in its online supplementary material. This material and code to reproduce the results in this study are available in the GitHub repository: https://github.com/rofa-g/lifert_mar.
