# Supplementary material for: Microsimulation reveals that medically assisted reproduction is unlikely to compensate for cohort fertility decline due to increasing maternal ages
Source: Hum Reprod. 2026 Feb 18;41(4):552–62. doi: 10.1093/humrep/deag006 (PMC13061122; doi:10.1093/humrep/deag006)
Supplement: deag006_Supplementary_Figure_S1 [file deag006_supplementary_figure_s1.pdf]

Human Reproduction, 2026, 1–1
https://doi.org/10.1093/humrep/deag006
Supplementary Data

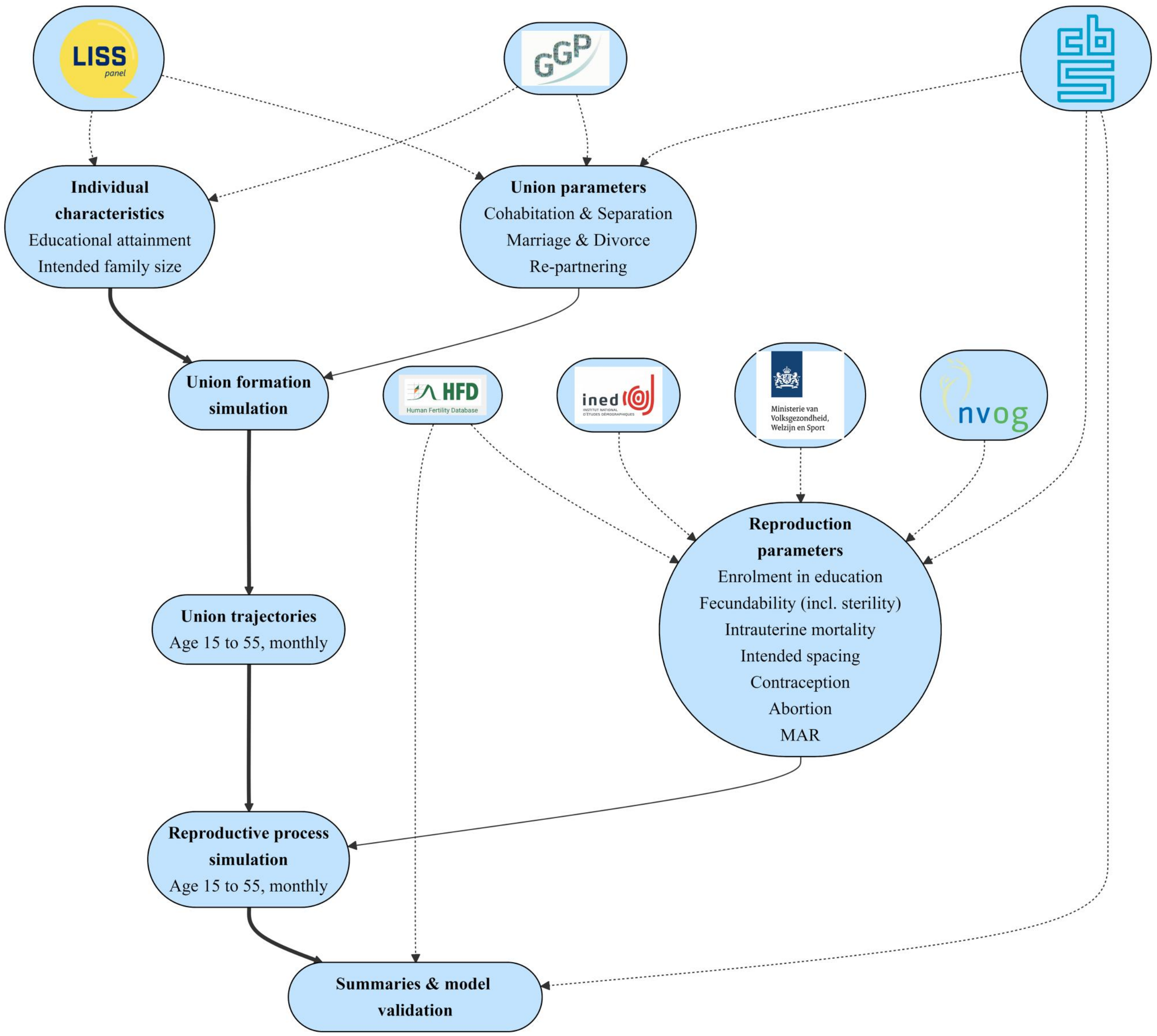

Supplementary Figure S1. **Overview of the simulation process**. The different logos indicate data sources. GGP, Generations and Gender Project (Generations and Gender Surveys); LISS, Longitudinal Internet studies for the Social Sciences; CBS, Statistics Netherlands; MAR, medically assisted reproduction.
