# Supplementary material for: Microsimulation reveals that medically assisted reproduction is unlikely to compensate for cohort fertility decline due to increasing maternal ages
Source: Hum Reprod. 2026 Feb 18;41(4):552–62. doi: 10.1093/humrep/deag006 (PMC13061122; doi:10.1093/humrep/deag006)
Supplement: deag006_Supplementary_Figure_S2 [file deag006_supplementary_figure_s2.pdf]

Human Reproduction, 2026, 1–1
https://doi.org/10.1093/humrep/deag006
Supplementary Data

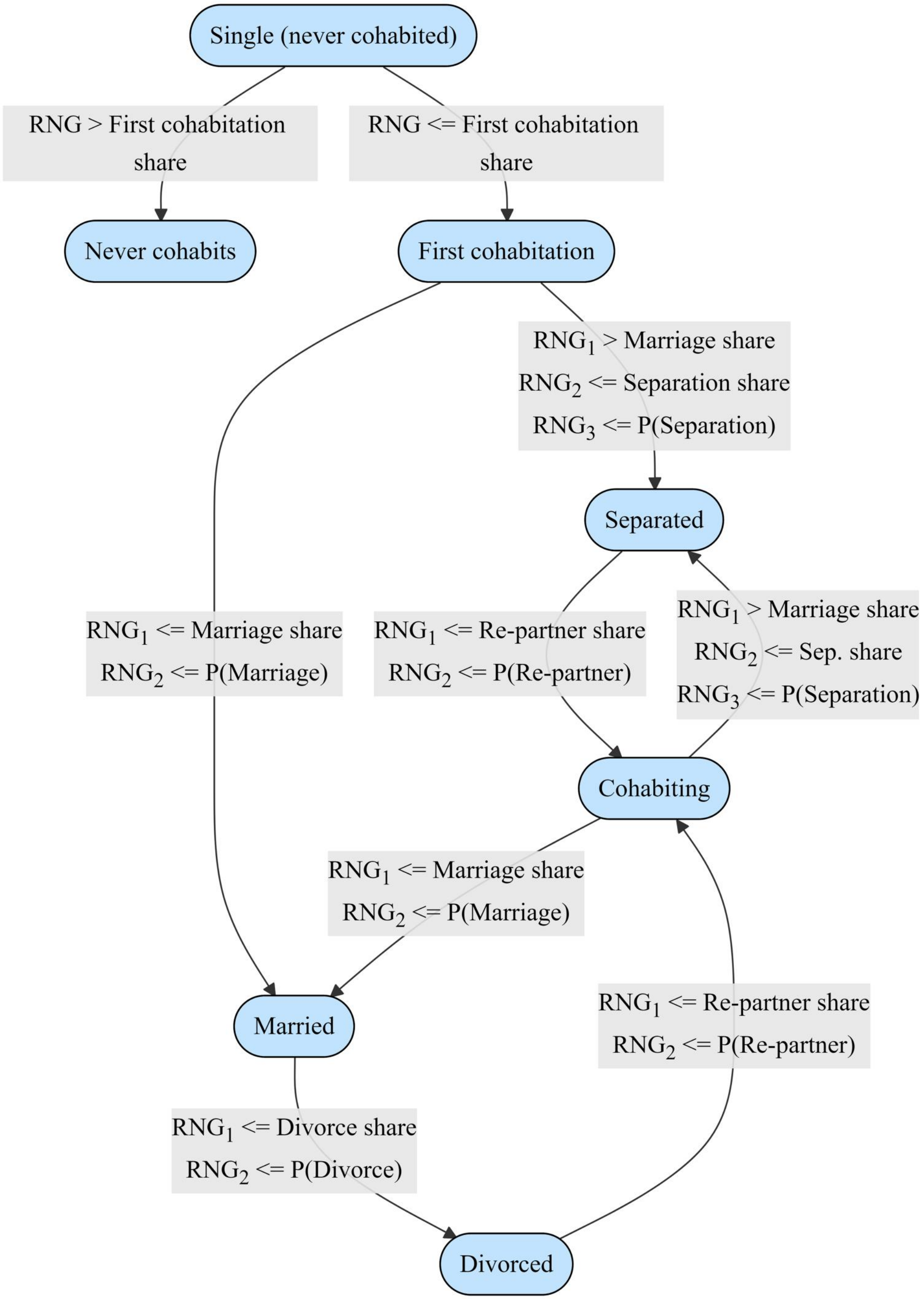

Supplementary Figure S2. Union formation simulation. RNG refers to a pseudo-randomly generated number between 0 and 1, which is compared to a cumulative distribution function. 'P()' denotes the probability of whatever is within the brackets. The month in which the randomly generated number meets the specified transition condition(s) the event occurs. If the transition conditions are not met, the woman remains in her current state.
