# Supplementary material for: Microsimulation reveals that medically assisted reproduction is unlikely to compensate for cohort fertility decline due to increasing maternal ages
Source: Hum Reprod. 2026 Feb 18;41(4):552–62. doi: 10.1093/humrep/deag006 (PMC13061122; doi:10.1093/humrep/deag006)
Supplement: deag006_Supplementary_Figure_S3 [file deag006_supplementary_figure_s3.pdf]

OXFORD
human reproduction

Human Reproduction, 2026, 1–1
https://doi.org/10.1093/humrep/deag006
Supplementary Data

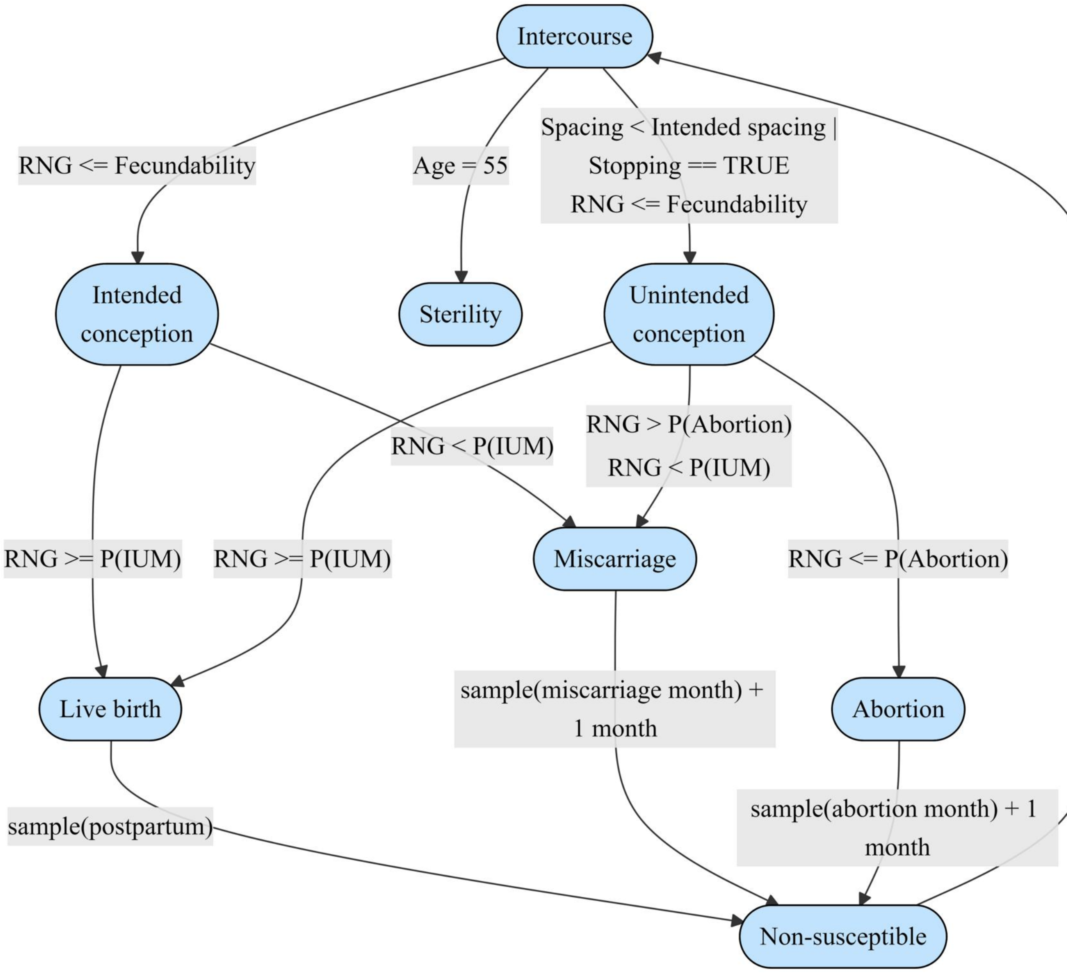

Supplementary Figure S3. Simulation of the reproductive process (while cohabiting or married). Sterility is an absorbing state, and ends the simulation. The arrows from live birth, intrauterine mortality, and abortion to non-susceptible denote the time spent in the state of non-susceptibility. Stopping is when the woman has reached her intended family size, spacing is the period during which the woman is not yet trying to get pregnant. Abortion here refers to (medically) induced abortion. ‘sample()’ refers to random sampling from the distribution within the brackets. TRUE refers to a Boolean ‘true’ logical statement. IUM, intrauterine mortality. RNG refers to a pseudo-randomly generated number between 0 and 1. ‘P()’ denotes the probability of whatever is within the brackets.
