# Supplementary material for: Microsimulation reveals that medically assisted reproduction is unlikely to compensate for cohort fertility decline due to increasing maternal ages
Source: Hum Reprod. 2026 Feb 18;41(4):552–62. doi: 10.1093/humrep/deag006 (PMC13061122; doi:10.1093/humrep/deag006)
Supplement: deag006_Supplementary_Figure_S4 [file deag006_supplementary_figure_s4.pdf]

Human Reproduction, 2026, 1–1
https://doi.org/10.1093/humrep/deag006
Supplementary Data

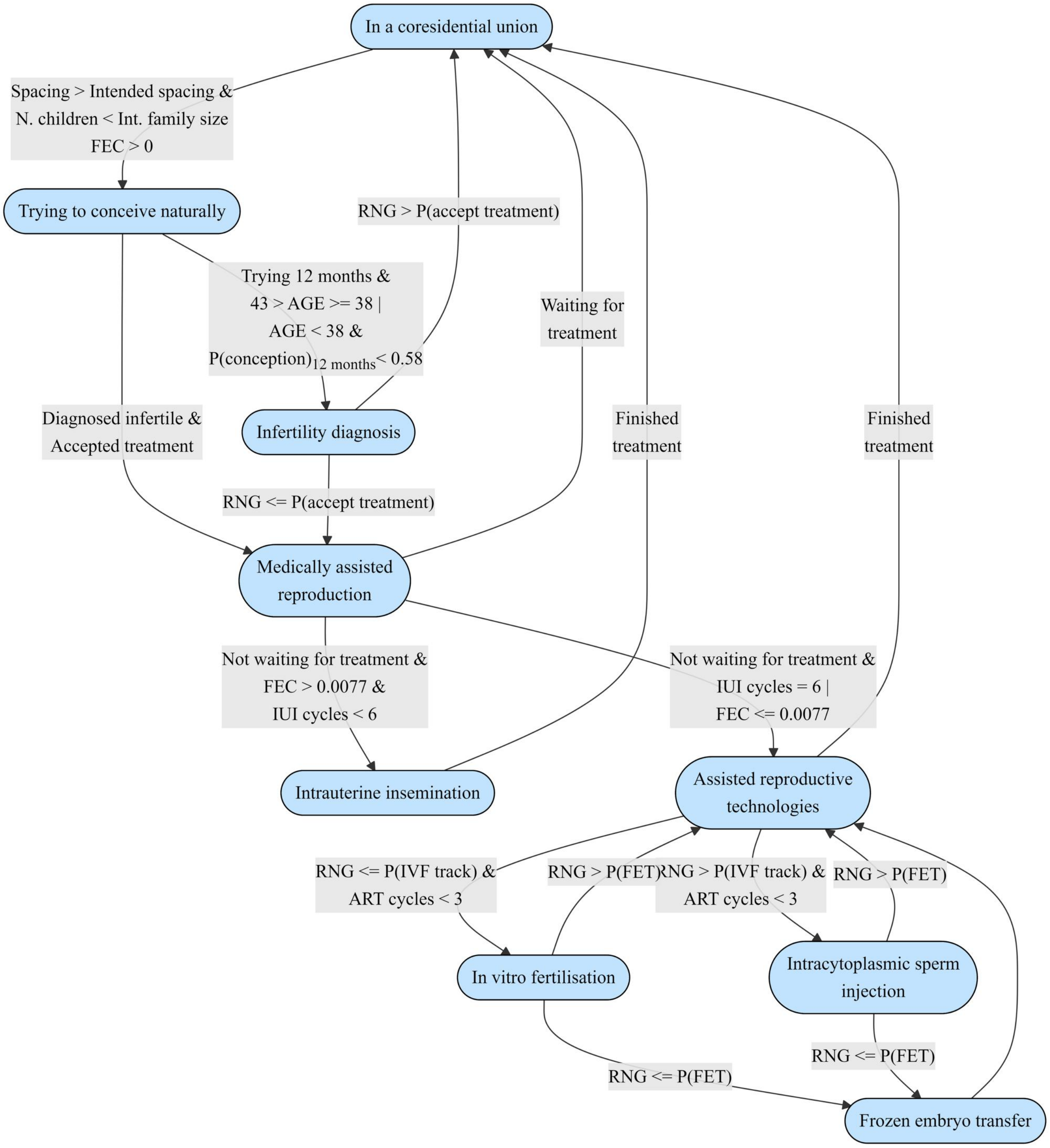

Supplementary Figure S4. Medically assisted reproduction path diagram. FEC, fecundability; FET, frozen embryo transfer. RNG refers to a pseudo-randomly generated number between 0 and 1. ‘P()’ denotes the probability of whatever is within the brackets. ‘$P(conception)_{12\ months}$’ is the probability of conceiving naturally within 12 months into the future.
