# Supplementary material for: Microsimulation reveals that medically assisted reproduction is unlikely to compensate for cohort fertility decline due to increasing maternal ages
Source: Hum Reprod. 2026 Feb 18;41(4):552–62. doi: 10.1093/humrep/deag006 (PMC13061122; doi:10.1093/humrep/deag006)
Supplement: deag006_Supplementary_Table_S1 [file deag006_supplementary_table_s1.pdf]

OXFORD
human reproduction
Human Reproduction, 2026, 1–1
https://doi.org/10.1093/humrep/deag006
Supplementary Data

Supplementary Table S1. Description of the data and parameters.

Parameters	Sample (size)	Processing/information	Format
Fecundability	–	Sampling from 10k nonparametric bootstrapped parameters from beta distribution beta distribution (a = 3, b = 9, mean ≈ 0.25) assuming a sample size of 1000. 12.5 years of linear decline before age at sterility (Leridon 2004, Leridon and Shapiro, 2017)	Pseudo-randomly generated from distribution
Intrauterine mortality	–	Fitted third degree polynomial distribution to values estimated by Leridon (1977, pp. 61–66), Month of miscarriage distribution based on clinical data[1]	Same distribution for all women, sampling from month of miscarriage distribution
Non-susceptible period	–	Live birth: truncated normal distribution (mean = 4, lower = 0)[2], miscarriage and abortion[3]: 1 month	Live birth: sampling from distribution, miscarriage and abortion: same for all women
Age at permanent sterility	–	Cubic spline interpolation between yearly data points from Leridon and Shapiro (2017), sampling from distribution	Sampling from distribution
Intended spacing	–	9 months of pregnancy + 5 months[4] waiting subtracted from the difference between the mean age at first cohabitation and the mean age at first birth, and HFD birth interval differences	Same distribution for all women
Contraception	–	spacing: 98.2% efficacy ages 15–25, 95.1% ages 26–55[5] stopping: 99.4% efficacy loosely based on Leridon and Shapiro (2017)	Same for all women
Unintended pregnancy	–	Unintended pregnancies ≈ 20%[6]	Same for all women
Medically induced abortion	–	60% probability, abortion ratio (154 abortions per 1000 live births, years 2000–2020) and month of pregnancy in which abortion occurred set to match official statistics[7]	Same for all women
Intended family size	1099	Intended family size recorded at ages 19–29 (GGS I, 2003) and 24–34 (LISS Family and Household wave 1, 2008).	Sampling from data
First cohabitation (and cohabitation)	ISCED 0–2: 168, ISCED 3–4: 469, ISCED 5–8: 651	Sampling from 10k nonparametric bootstrapped parameters from original data assuming Gumbel distribution (best fit to data, based on AIC), cohort share who ever cohabit 95% based on 1954–1964 GGS cohort and Bellani *et al.* (2017)[8], education specific shares calculated with equation systems from the ratios between the educational shares of the 1964–1984 GGS cohort[9] that sum up to the cohort share, share who stay cohabited after first cohabitation 10%[10]	RNG compared with CDF value corresponding to the iteration/month
Cohabitation to marriage	ISCED 0–2: 309, ISCED 3–4: 798, ISCED 5–8: 705	Sampling from 10k nonparametric bootstrapped parameters from original data assuming exponential distribution (best fit to data, based on AIC), cohort share who marry of 58.0% based on CBS reports[10], 70% share who ever married based on CBS estimates[11], education specific shares estimated the same way as	—″—

(continued)

Supplementary Table S1. (continued)

Parameters	Sample (size)	Processing/information	Format
		first cohabitation, but with 1954–1984 cohort[12]	
Separation	724	Sampling from 10k nonparametric bootstrapped parameters from original data assuming exponential distribution (best fit to data, based on AIC), cohort share who separate of 31.2% based on CBS report[13], education specific shares estimated the same way as first cohabitation, but with 1954–1974 cohort[12]	—″—
Re-partnering	250	Sampling from 10k nonparametric bootstrapped parameters from original data assuming Gumbel distribution (best fit to data, based on AIC), Re-partnering set at 75%, based on Finnish 1969–1971 birth cohort (76.2%)[14], education specific shares estimated the same way as first cohabitation, but with 1954–1964 cohort[12]	—″—
Divorce	496	Sampling from 10k nonparametric bootstrapped parameters from original data assuming exponential distribution (best fit to data, based on AIC), cohort share who divorce of 27.6% based on CBS report[15] (separate + marry*divorce ≈ 48%)[9], education specific shares estimated the same way as first cohabitation, but with 1954–1964 cohort[11]	—″—
Education	ISCED 0–2: 357, ISCED 3–4: 1007, ISCED 5–8: 1209	Educational structure from LISS and GGS, duration of enrolment based on expected year of graduation	Educational attainment sampled from distribution
Share of births outside of coresidential unions	National population register	Approximate mean between the years 1996, 2011, and 2022 using data from CBS[16]	Reference number for comparison with simulation output
Share of eligible women who undergo MAR	National ART registers, surveys	Mean ART share of total births 2003–2020 was 2.4%[17], IUI share of all births approximated at 1.3% based on Danish data[18], the probability that an eligible woman takes up MAR treatment is based on nonparametric bootstrapped values using the mean share and sample size from[17]	RNG compared with single parameter for the probability that a woman undergoes MAR treatment
Probabilities of twin and triplet births	National population register, National ART register	Age specific probability of multiple birth[19] multiplied by ratio of MAR[20] to natural multiple birth[21]	RNG compared with CDF value corresponding to the iteration/month, twin or triplet draw based on ratio between twin and triplet births
MAR pregnancy rates	National ART registers, combined clinic records (IUI)	Age specific pregnancy rates for IVF/ICSI[22] multiplied by ratio of aggregate pregnancy rates between IVF and ICSI, FET, and IUI[23]	RNG compared with CDF value corresponding to the iteration/month
Number of cycles	–	Six cycles of IUI, followed by three cycles of IVF/ICSI based on basic health insurance coverage[24]	Single parameters compared with cycle counts in simulation
Maximum age at treatment	–	Below 43 years of age based on basic health insurance coverage[25]	Single parameter compared with woman's current age
MAR treatment path	–	The threshold for MAR uptake set to <58% probability of conception within 12 months of infertility diagnosis for women up to age 38[26], fecundability threshold of <0.0077 for starting ART (all ages) using fecundability distribution of Leridon and Shapiro (2017)[27], IVF or ICSI	Single parameters compared with cycle counts, age, and fecundability thresholds

(continued)

Supplementary Table S1. (continued)

Parameters	Sample (size)	Processing/information	Format
		based on real ratio (number of cycles) between the two[21]	
Waiting time to/between treatments	–	Waiting time before and between MAR treatments adjusted to match assumptions in van Eekelen *et al.* (2020)[28] with four IUI cycles per year (based on Custers *et al.* (2007)[29] and van Eekelen *et al.* (2019)[30]) and 1.5 IVF/ICSI cycles per year (based on Eijkemans *et al.* (2017)[31] and van Eekelen *et al.* 2019[32])	IVF/ICSI: 7 months of waiting before treatment, and 7 months of waiting between treatments IUI: 1 months waiting before treatment, 2 months of waiting between treatments

RNG, random number generator; CDF, cumulative distribution function; GGS, Generations and Gender Survey; LISS, Longitudinal Internet studies for the Social Sciences; CBS, Statistics Netherlands; HFD, Human Fertility Database; MAR, medically assisted reproduction; AIC, Akaike information criterion. [1](Wilcox *et al.*, 1988; Dugas and Slane, 2023), [2](Leridon, 1977; Theurich *et al.*, 2019), [3](Donnet *et al.*, 1990; Schreiber *et al.*, 2011),[4] 5 months based on a mean fecundability of 0.25 using the formula in Leridon (1977 page 27), [5](CBS, 2014), [6](Levels *et al.*, 2012; Bearak *et al.*, 2022), [7](MVWS, 2017, 2021), **[8]**(Bellani *et al.*, 2017), **[9]**Cohort range expanded to reach sufficient sample sizes to fit distributions, [10](CBS, 2019), **[11]**(Stoeldrajer *et al.*, 2021), [12]Cohort ranges chosen based on the average age at the different union events. The 1974–1984 cohort could not be used because this cohort was too young at the time of the survey to have reliable retrospective data on past union events (especially for highly educated women), [13](Kooiman *et al.*, 2021), [14]Ever repartnered/(Never repartnered and separated + Ever repartnered). Data are for both sexes combined (Andersson *et al.*, 2022), [15](Kooiman, 2022), [16](CBS, 2023a), [17]ART births as a share of total births 2003, 2010, and 2020 (CBS, 2023b; Stichting LIR, 2024), [18]Danish (2013 and 2018) (DST, 2024; Sundhedsdatastyrelsen, 2024) and Dutch using 2019 ART shares of total births as a proxy for MAR use between Denmark and the Netherlands (CBS, 2023b; Stichting LIR, 2024), [19](van der Steeg *et al.*, 2007), [20]mean between 1995, 2005, and 2015 (CBS, 2016), [21]mean between 2003, 2010, and 2020 (Stichting LIR, 2024), for IUI no Dutch data; mean based on Danish data from 2007 and 2018 (de Mouzon *et al.*, 2012; Wyns *et al.*, 2022), [22]National birth register (CBS, 2023b), [23](Habbema *et al.*, 2015), [24]2013 aggregate ART pregnancy rates (Stichting LIR, 2024) and IUI aggregate pregnancy rate from 2013 Danish data (Sundhedsdatastyrelsen, 2024), [25](MVWS, 2022), [26](Hunault *et al.*, 2005; van der Steeg *et al.*, 2007), [27](Leridon and Shapiro, 2017), [28](van Eekelen *et al.*, 2020), [29](Custers *et al.*, 2007), [30](van Eekelen *et al.*, 2019b), [31](Eijkemans *et al.*, 2017), [32](van Eekelen *et al.*, 2019a).

References

Andersson L, Jalovaara M, Uggla C, Saarela J. Less is more? Repartnering and completed cohort fertility in Finland. *Demography* 2022;59:2321–2339.
Bearak JM, Popinchalk A, Beavin C, Ganatra B, Moller A-B, Tunçalp Ö, Alkema L. Country-specific estimates of unintended pregnancy and abortion incidence: a global comparative analysis of levels in 2015–2019. *BMJ Glob Health* 2022;7:e007151.
Bellani D, Esping-Andersen G, Nedoluzhko L. Never partnered: a multilevel analysis of lifelong singlehood. *Demogr Res* 2017;37:53–100.
CBS. Gebruik pil daalt, spiraaltje wint terrein. *Cent Bur Voor Stat* 2014. https://www.cbs.nl/nl-nl/nieuws/2014/25/gebruik-pil-daalt-spiraaltje-wint-terrein
CBS. Minder tweelingen geboren. *Cent Bur Voor Stat* 2016. https://www.cbs.nl/nl-nl/nieuws/2016/39/minder-tweelingen-geboren
CBS. Half of cohabitants still together after 15 years. *Stat Neth* 2019. https://www.cbs.nl/en-gb/news/2019/06/half-of-cohabitants-still-together-after-15-years
CBS. How many babies were born to unmarried mothers? – The Netherlands in numbers. *CBS* 2023a. https://longreads.cbs.nl/the-netherlands-in-numbers-2023/how-many-babies-were-born-to-unmarried-mothers
CBS. StatLine—Geboorte; kerncijfers, 1950-2022. *Cent Bur Voor Stat* 2023b. https://opendata.cbs.nl/statline/#/CBS/nl/dataset/37422NED/table? fromstatweb
Custers IM, Steures P, van der Steeg JW, van Dessel TJHM, Bernardus RE, Bourdrez P, Koks CAM, Riedijk WJ, Burggraaff JM, van der Veen F, *et al.* External validation of a prediction model for an ongoing pregnancy after intrauterine insemination. *Fertil Steril* 2007;88:425–431.
de Mouzon J, Goossens V, Bhattacharya S, Castilla JA, Ferraretti AP, Korsak V, Kupka M, Nygren KG, Andersen AN. Assisted reproductive technology in Europe, 2007: results generated from European registers by ESHRE. *Hum Reprod* 2012;27:954–966.
Donnet ML, Howie PW, Marnie M, Cooper W, Lewis M. Return of ovarian function following spontaneous abortion. *Clin Endocrinol (Oxf)* 1990;33:13–20.
DST. Births. *Stat Den* 2024. https://www.dst.dk/en/Statistik/emner/borgere/befolkning/foedsler
Dugas C, Slane VH. Miscarriage. *StatPearls* 2023. http://www.ncbi.nlm.nih.gov/books/NBK532992/
Eijkemans MJC, Kersten FAM, Lintsen AME, Hunault CC, Bouwmans CAM, Roijen LH, Habbema JDF, Braat DDM. Cost-effectiveness of ‘immediate IVF’ versus ‘delayed IVF’: a prospective study. *Hum Reprod* 2017;32:999–1008.
Habbema JDF, Eijkemans MJC, Leridon H, te Velde ER. Realizing a desired family size: when should couples start? *Hum Reprod* 2015;30:2215–2221.
Hunault CC, Laven JSE, van Rooij IAJ, Eijkemans MJC, te Velde ER, Habbema JDF. Prospective validation of two models predicting pregnancy leading to live birth among untreated subfertile couples. *Hum Reprod* 2005;20:1636–1641.
Kooiman N. Trends in (echt)scheidingen [Internet]. Centraal Bureau voor de Statistiek, 2022. https://www.cbs.nl/nl-nl/longread/statistische-trends/2022/trends-in-echt-scheidingen? onepage=true
Kooiman N, Stoeldrajer L, Harmsen C. Huidige twintigers gaan vaker uit elkaar. 2021. https://www.cbs.nl/nl-nl/longread/statistische-trends/2021/huidige-twintigers-gaan-vaker-uit-elkaar? onepage=true
Leridon H. Human fertility: the basic components. Chicago: University of Chicago Press, 1977.
Leridon H, Shapiro D. Biological effects of first birth postponement and assisted reproductive technology on completed fertility. *Popul Engl Ed* 2002-2017;72:445–472.
Levels M, Need A, Nieuwenhuis R, Sluiter R, Ultee W. Unintended pregnancy and induced abortion in the Netherlands 1954–2002. *Eur Sociol Rev* 2012;28:301–318.
MVWS. Jaarrapportage Wet afbreking zwangerschap (Wafz) 2015. Ministerie van Volksgezondheid, Welzijn en Sport, 2017. https://zoek.officielebekendmakingen.nl/blg-799078.pdf
MVWS. Jaarrapportage 2020 Wet afbreking zwangerschap (Wafz). Ministerie van Volksgezondheid, Welzijn en Sport, 2021. https://open.overheid.nl/repository/ronl-d18f3b07-782e-4b08-99c6-675b0d60ca33/1/pdf/jaarrapportage-2020-wet-afbreking-zwangerschap-wafz.pdf
MVWS. Vruchtbaarheidsbehandeling (Zvw) – Verzekerde zorg—Zorginstituut Nederland. Ministerie van Volksgezondheid, Welzijn en Sport, 2022. https://www.zorginstituutnederland.nl/Verzekerde+zorg/vruchtbaarheidsbehandeling-zvw
Schreiber CA, Sober S, Ratcliffe S, Creinin MD. Ovulation resumption after medical abortion with mifepristone and misoprostol. *Contraception* 2011;84:230–233.
Stichting LIR. Landelijke IVF-cijfers. Nederlandse Vereigning voor Obsterie en Gynaecologie, 2024. https://www.degynaecoloog.nl/nuttige-informatie/ivf-resultaten/
Stoeldrajer L, te Riele S, van Duin C, van der Reijden P. Huishoudensprognose 2021-2070: Groei aantal huishoudens houdt aan. CBS 2021. https://www.cbs.nl/nl-nl/longread/statistische-trends/2021/huishoudensprognose-2021-2070-groei-aantal-huishoudens-houdt-aan/3-model-en-veronderstellingen
Sundhedsdatastyrelsen. Assisteret reproduktion—Sundhedsdatastyrelsen. *Dan Health Data Auth* 2024. https://sundhedsdatastyrelsen.dk/data-og-registre/publikationer/sygdomme-og-behandlinger/assisteret-reproduktion
Theurich MA, Davanzo R, Busck-Rasmussen M, Díaz-Gómez NM, Brennan C, Kylberg E, Bærug A, McHugh L, Weikert C, Abraham K, *et al.* Breastfeeding rates and programs in Europe: a survey of 11 national breastfeeding committees and representatives. *J Pediatr Gastroenterol Nutr* 2019;68:400.
van der Steeg JW, Steures P, Eijkemans MJC, Habbema JDF, Hompes PGA, Broekmans FJ, van Dessel HJHM, Bossuyt PMM, van der Veen F, Mol BWJ, *et al.* Pregnancy is predictable: a large-scale prospective external validation of the prediction of spontaneous pregnancy in subfertile couples. *Hum Reprod* 2007;22:536–542.
van Eekelen R, Eijkemans MJ, Mochtar M, Mol F, Mol BW, Groen H, van Wely M. Cost-effectiveness of medically assisted reproduction or expectant management for unexplained subfertility: when to start treatment? *Hum Reprod* 2020;35:2037–2046.
van Eekelen R, van Geloven N, van Wely M, Bhattacharya S, van der Veen F, Eijkemans MJ, McLernon DJ. IVF for unexplained subfertility; whom should we treat? *Hum Reprod* 2019a;34:1249–1259.
van Eekelen R, van Geloven N, van Wely M, McLernon DJ, Mol F, Custers IM, Steures P, Bhattacharya S, Mol BW, van der Veen F, *et al.* Is IUI with ovarian stimulation effective in couples with unexplained subfertility? *Hum Reprod* 2019b;34:84–91.
Wilcox AJ, Weinberg CR, O'Connor JF, Baird DD, Schlatterer JP, Canfield RE, Armstrong EG, Nisula BC. Incidence of early loss of pregnancy. *N Engl J Med* 1988;319:189–194.
Wyns C, De Geyter C, Calhaz-Jorge C, Kupka MS, Motrenko T, Smeenk J, Bergh C, Tandler-Schneider A, Rugescu IA, Goossens V. ART in Europe, 2018: results generated from European registries by ESHRE†. *Hum Reprod Open* 2022;2022:hoac022.
