# Supplementary material for: Microsimulation reveals that medically assisted reproduction is unlikely to compensate for cohort fertility decline due to increasing maternal ages
Source: Hum Reprod. 2026 Feb 18;41(4):552–62. doi: 10.1093/humrep/deag006 (PMC13061122; doi:10.1093/humrep/deag006)
Supplement: deag006_Supplementary_Table_S2 [file deag006_supplementary_table_s2.pdf]

OXFORD
human reproduction

Human Reproduction, 2026, 1–1
https://doi.org/10.1093/humrep/deag006
Supplementary Data

Supplementary Table S2. Completed cohort fertility with medically assisted reproduction of Dutch women born during 1974–1984.

Indicator	Demographic data	Simulation results (1974–1984 cohort)
Mean age at first cohabitation (GGS, LISS)	24.5	23.725
Mean age at first marriage (CBS, 2004–2014)[1]	29.7	28.589
Mean age at first separation, no previous divorce (GGS, LISS)	28.5	29.041
Mean age at first divorce (GGS, LISS)	37.6	37.579
Mean age at first repartnering		32.927
Percent ever cohabited (GGS, estimate)	95	95.047
Percent ever married (CBS, estimate)[2]	70	71.995
Percent cohabited and never separated or married (CBS, estimate)[3]	10	9.627
Percent marriage (cohabitation to marriage; CBS, estimate)[3]	58	57.340
Percent separation (CBS, estimate)[4]	32	31.280
Percent divorce (CBS, estimate)[5]	27.6	25.310
Percent repartnering (Finnish register data[6], estimate)	75	73.244
Mean age at first birth (HFD, 1979 cohort)[7]	29.2	28.838
Mean age second birth (HFD, 1979 cohort)[7]	31.6	31.338
Mean age third birth (HFD, 1979 cohort)[7]	33.1	33.113
Mean age fourth birth (HFD, 1979 cohort)[7]	34.5	34.585
Completed cohort fertility (within coresidential unions) (HFD, 1979 cohort)[7]	1.80 (1.66)	1.735
Fertility gap (within coresidential unions) (GGS, LISS, HFD)	0.227 (0.364)	0.290
0 children (HFD, 1969 cohort, %)	17.6	23.751
1 child (HFD, 1969 cohort, %)	18.5	10.717
2 children (HFD, 1969 cohort, %)	42.7	41.654
3 children (HFD, 1969 cohort, %)	15.6	17.626
4+ children (HFD, 1969 cohort, %)	5.6	6.252
Miscarriages per live birth		0.178
Percent unintended pregnancies[8]	20	19.680
Abortion ratio 2000–2020[9] (abortions per 1000 live births)	154	155.693
Percent of births outside coresidential union[10]	10	2.316
Percent IUI births[11]	1.3	1.341
Percent ART births[12]	2.4	2.390

GGS, Generations and Gender Survey; LISS, Longitudinal Internet studies for the Social Sciences; CBS, Statistics Netherlands; HFD, Human Fertility Database. The empty fields in the 'Demographic data' column for mean age at first repartnering and miscarriages per live birth are empty because there was no reference data available. A general note about the simulated mean ages at union events being younger than in the reference (raw) data. This is because distribution functions were fitted to the data, and the data did not fit any distribution very well, due to small samples and because the GGS data that we rely quite heavily on is old (2003), which means that it only captures the early half of the reproductive lives of the women in our sample. This likely contributed to the overestimation of the fertility gap in our simulation output. The overestimated share of childless women and underestimated share of women with one child compared to the reference data is because our model is missing 7.7 percentage points (10–2.3 percentage points) of births that occur outside coresidential unions. Assuming that these missing births were first births (argument in the model validation section of Granholm *et al.*, 2025), the share of childless women produced by our model would have decreased to 16.1% and the share of women with 1 child would have increased to 18.4%, which are close to the reference values. [1] Mean age at first marriage estimated by calculating the difference in the mean age at first marriage for 2022 (36.4–32.8 = 3.6), subtracting this difference from the mean ages at marriage 2004–2014, and taking the mean of those means. 2004–2014 was chosen to roughly cover the age at first marriage range (ages 30–40 years) for the simulated 1974–1984 cohort. [2] (Stoeldrajer *et al.*, 2021), [3] (CBS, 2019), [4] (Kooiman *et al.*, 2021), [5] (Kooiman, 2022), [6] Ever repartnered/(Never repartnered and separated + Ever repartnered). Data are for both sexes combined (Andersson *et al.*, 2022), [7] The mean ages at birth and completed cohort fertility are approximations based on means for women aged 40 years, as the 1979 cohort has not yet completed its fertility. The approximation is the trend in the difference between means for women who had completed their fertility and women aged 40 years for the 1959–1969 birth cohorts. [8] (Bakker *et al.*, 2009; Levels *et al.*, 2012), [9] (MVWS, 2017, 2021), [10] Approximated based on CBS (2023a), [11] Mean share of ART births over the years 2003, 2010, and 2020 (CBS, 2023b; Stichting LIR, 2024), [12] Mean share of IUI births over the years 2013 and 2018 using Danish data as proxy (DST, 2024; Sundhedsdatastyrelsen, 2024).

References

Andersson L, Jalovaara M, Uggla C, Saarela J. Less is more? Repartnering and completed cohort fertility in Finland. *Demography* 2022;59:2321–2339.
Bakker F, de Graaf H, de Haas S, Kedde H, Kruijer H, Wijsen C. Seksuele gezondheid in Nederland 2009. Utrecht: Rutgers Nisso Groep, 2009. https://docplayer.nl/698192-Seksuele-gezondheid-in-nederland-2009.html
CBS. Half of cohabitants still together after 15 years. *Stat Neth* 2019. https://www.cbs.nl/en-gb/news/2019/06/half-of-cohabitants-still-together-after-15-years
CBS. How many babies were born to unmarried mothers? – The Netherlands in numbers. *CBS* 2023a. https://longreads.cbs.nl/the-netherlands-in-numbers-2023/how-many-babies-were-born-to-unmarried-mothers
CBS. StatLine—Geboorte; kerncijfers, 1950-2022. *Cent Bur Voor Stat* 2023b. https://opendata.cbs.nl/statline/#/CBS/nl/dataset/37422NED/table? fromstatweb
DST. Births. *Stat Den* 2024. https://www.dst.dk/en/Statistik/emner/borgere/befolkning/foedsler
Granholm R, Gauthier A, Stulp G. Examining the relationships between education, coresidential unions, and the fertility gap by simulating the reproductive life courses of Dutch women. *Demogr Res* 2025;52:797–848.
Kooiman N. Trends in (echt)scheidingen. Centraal Bureau voor de Statistiek, 2022. https://www.cbs.nl/nl-nl/longread/statistische-trends/2022/trends-in-echt-scheidingen? onepage=true
Kooiman N, Stoeldrajer L, Harmsen C. Huidige twintigers gaan vaker uit elkaar. 2021. https://www.cbs.nl/nl-nl/longread/statistische-trends/2021/huidige-twintigers-gaan-vaker-uit-elkaar? onepage=true
Levels M, Need A, Nieuwenhuis R, Sluiter R, Ultee W. Unintended pregnancy and induced abortion in the Netherlands 1954–2002. *Eur Sociol Rev* 2012;28:301–318.
MVWS. Jaarrapportage Wet afbreking zwangerschap (Wafz) 2015. Ministerie van Volksgezondheid, Welzijn en Sport, 2017. https://zoek.officielebekendmakingen.nl/blg-799078.pdf
MVWS. Jaarrapportage 2020 Wet afbreking zwangerschap (Wafz) [Internet]. Ministerie van Volksgezondheid, Welzijn en Sport, 2021. https://open.overheid.nl/repository/ronl-d18f3b07-782e-4b08-99c6-675b0d60ca33/1/pdf/jaarrapportage-2020-wet-afbreking-zwangerschap-wafz.pdf
Stichting LIR. Landelijke IVF-cijfers. Nederlandse Vereigning voor Obsterie en Gynaecologie, 2024. https://www.degynaecoloog.nl/nuttige-informatie/ivf-resultaten/
Stoeldrajer L, te Riele S, van Duin C, van der Reijden P. Huishoudensprognose 2021-2070: Groei aantal huishoudens houdt aan. CBS 2021. https://www.cbs.nl/nl-nl/longread/statistische-trends/2021/huishoudensprognose-2021-2070-groei-aantal-huishoudens-houdt-aan/3-model-en-veronderstellingen
Sundhedsdatastyrelsen. Assisteret reproduktion—Sundhedsdatastyrelsen. *Dan Health Data Auth* 2024. https://sundhedsdatastyrelsen.dk/data-og-registre/publikationer/sygdomme-og-behandlinger/assisteret-reproduktion
