# Supplementary material for: Microsimulation reveals that medically assisted reproduction is unlikely to compensate for cohort fertility decline due to increasing maternal ages
Source: Hum Reprod. 2026 Feb 18;41(4):552–62. doi: 10.1093/humrep/deag006 (PMC13061122; doi:10.1093/humrep/deag006)
Supplement: deag006_Supplementary_Table_S3 [file deag006_supplementary_table_s3.pdf]

OXFORD
human reproduction
Human Reproduction, 2026, 1–1
https://doi.org/10.1093/humrep/deag006
Supplementary Data

Supplementary Table S3. Completed cohort fertility without medically assisted reproduction of Dutch women born during 1974–1984.

Indicator	Demographic data	Simulation results (1974–1984 cohort)
Mean age at first cohabitation (GGS, LISS)	24.5	23.721
Mean age at first marriage (CBS, 2004–2014)[1]	29.7	28.577
Mean age at first separation, no previous divorce (GGS, LISS)	28.5	29.060
Mean age at first divorce (GGS, LISS)	37.6	37.557
Mean age at first repartnering		32.925
Percent ever cohabited (GGS, estimate)	95	94.978
Percent ever married (CBS, estimate)[2]	70	71.974
Percent cohabited and never separated or married (CBS, estimate)[3]	10	9.612
Percent marriage (cohabitation to marriage; CBS, estimate)[3]	58	57.366
Percent separation (CBS, estimate)[4]	32	31.291
Percent divorce (CBS, estimate)[5]	27.6	25.234
Percent repartnering (Finnish register data[6], estimate)	75	73.248
Mean age at first birth (HFD, 1979 cohort)[7]	29.2	28.760
Mean age second birth (HFD, 1979 cohort)[7]	31.6	31.253
Mean age third birth (HFD, 1979 cohort)[7]	33.1	33.022
Mean age fourth birth (HFD, 1979 cohort)[7]	34.5	34.437
Completed cohort fertility (within coresidential unions) (HFD, 1979 cohort)[7]	1.80 (1.66)	1.692
Fertility gap (within coresidential unions) (GGS, LISS, HFD)	0.227 (0.364)	0.333
0 children (HFD, 1969 cohort, %)	17.6	25.042
1 child (HFD, 1969 cohort, %)	18.5	10.989
2 children (HFD, 1969 cohort, %)	42.7	40.979
3 children (HFD, 1969 cohort, %)	15.6	17.120
4+ children (HFD, 1969 cohort, %)	5.6	5.870
Miscarriages per live birth		0.175
Percent unintended pregnancies[8]	20	20.111
Abortion ratio 2000–2020[9] (abortions per 1000 live births)	154	159.857
Percent of births outside coresidential union[10]	10	2.337
Percent IUI births[11]	0	0.000
Percent ART births[12]	0	0.000

GGS, Generations and Gender Survey; LISS, Longitudinal Internet studies for the Social Sciences; CBS, Statistics Netherlands; HFD, Human Fertility Database. The empty fields in the 'Demographic data' column for mean age at first repartnering and miscarriages per live birth are empty because there was no reference data available. A general note about the simulated mean ages at union events being younger than in the reference (raw) data. This is because distribution functions were fitted to the data, and the data did not fit any distribution very well, due to small samples and because the GGS data that we rely quite heavily on is old (2003), which means that it only captures the early half of the reproductive lives of the women in our sample. This likely contributed to the overestimation of the fertility gap in our simulation output. [1] Mean age at first marriage estimated by calculating the difference in the mean age at first marriage for 2022 (36.4–32.8 = 3.6), subtracting this difference from the mean ages at marriage 2004–2014, and taking the mean of those means. 2004–2014 was chosen to roughly cover the age at first marriage range (ages 30–40 years) for the simulated 1974–1984 cohort. [2] (Stoeldrajer *et al.*, 2021), [3](van Gaalen *et al.*, 2019), [4] (Kooiman *et al.*, 2021), [5] (Kooiman, 2022), [6] Ever repartnered/(Never repartnered and separated + Ever repartnered). Data are for both sexes combined (Andersson *et al.*, 2022), [7] The mean ages at birth and completed cohort fertility are approximations based on means for women aged 40 years, as the 1979 cohort has not yet completed its fertility. The approximation is the trend in the difference between means for women who had completed their fertility and women aged 40 years for the 1959–1969 birth cohorts. [8] (Bakker *et al.*, 2009; Levels *et al.*, 2012), [9] (MVWS, 2017, 2021), [10] Approximated based on CBS (2023a), [11] Mean share of ART births over the years 2003, 2010, and 2020 (CBS, 2023b; Stichting LIR, 2024), [12] Mean share of IUI births over the years 2013 and 2018 using Danish data as proxy (DST, 2024; Sundhedsdatastyrelsen, 2024).

References

Andersson L, Jalovaara M, Uggla C, Saarela J. Less is more? Repartnering and completed cohort fertility in Finland. *Demography* 2022;59:2321–2339.
Bakker F, de Graaf H, de Haas S, Kedde H, Kruijer H, Wijsen C. Seksuele gezondheid in Nederland 2009. Utrecht: Rutgers Nisso Groep, 2009. https://docplayer.nl/698192-Seksuele-gezondheid-in-nederland-2009.html
CBS. How many babies were born to unmarried mothers? – The Netherlands in numbers. *CBS* 2023a. https://longreads.cbs.nl/the-netherlands-in-numbers-2023/how-many-babies-were-born-to-unmarried-mothers
CBS. StatLine—Geboorte; kerncijfers, 1950-2022. *Cent Bur Voor Stat* 2023b. https://opendata.cbs.nl/statline/#/CBS/nl/dataset/37422NED/table? fromstatweb
DST. Births. *Stat Den* 2024. https://www.dst.dk/en/Statistik/emner/borgere/befolkning/foedsler
Kooiman N. Trends in (echt)scheidingen. Centraal Bureau voor de Statistiek, 2022. https://www.cbs.nl/nl-nl/longread/statistische-trends/2022/trends-in-echt-scheidingen? onepage=true
Kooiman N, Stoeldraijer L, Harmsen C. Huidige twintigers gaan vaker uit elkaar. Centraal Bureau voor de Statistiek, 2021. https://www.cbs.nl/nl-nl/longread/statistische-trends/2021/huidige-twintigers-gaan-vaker-uit-elkaar? onepage=true
Levels M, Need A, Nieuwenhuis R, Sluiter R, Ultee W. Unintended pregnancy and induced abortion in the Netherlands 1954–2002. *Eur Sociol Rev* 2012;28:301–318.
MVWS. Jaarrapportage Wet afbreking zwangerschap (Wafz) 2015 [Internet]. Ministerie van Volksgezondheid, Welzijn en Sport, 2017. https://zoek.officielebekendmakingen.nl/blg-799078.pdf
MVWS. Jaarrapportage 2020 Wet afbreking zwangerschap (Wafz) [Internet]. Ministerie van Volksgezondheid, Welzijn en Sport, 2021. https://open.overheid.nl/repository/ronl-d18f3b07-782e-4b08-99c6-675b0d60ca33/1/pdf/jaarrapportage-2020-wet-afbreking-zwangerschap-wafz.pdf
Stichting LIR. Landelijke IVF-cijfers [Internet]. Nederlandse Vereigning voor Obsterie en Gynaecologie, 2024. https://www.degynaecoloog.nl/nuttige-informatie/ivf-resultaten/
Stoeldrajer L, te Riele S, van Duin C, van der Reijden P. Huishoudensprognose 2021-2070: Groei aantal huishoudens houdt aan. Centraal Bureau voor de Statistiek, 2021. https://www.cbs.nl/nl-nl/longread/statistische-trends/2021/huishoudensprognose-2021-2070-groei-aantal-huishoudens-houdt-aan
Sundhedsdatastyrelsen. Assisteret reproduktion—Sundhedsdatastyrelsen. *Dan Health Data Auth* 2024. https://sundhedsdatastyrelsen.dk/data-og-registre/publikationer/sygdomme-og-behandlinger/assisteret-reproduktion
van Gaalen R, van Houdt K, Poortman A-R. Trouwen, kinderen krijgen en scheiden naar opleiding. 2019. https://www.cbs.nl/nl-nl/longread/statistische-trends/2019/trouwen-kinderen-krijgen-en-scheiden-naar-opleiding? onepage=true
