# Supplementary material for: Microsimulation reveals that medically assisted reproduction is unlikely to compensate for cohort fertility decline due to increasing maternal ages
Source: Hum Reprod. 2026 Feb 18;41(4):552–62. doi: 10.1093/humrep/deag006 (PMC13061122; doi:10.1093/humrep/deag006)
Supplement: deag006_Supplementary_Table_S4 [file deag006_supplementary_table_s4.pdf]

Human Reproduction, 2026, 1–1
https://doi.org/10.1093/humrep/deag006
Supplementary Data

Supplementary Table S4. Completed cohort fertility with medically assisted reproduction by level of education of Dutch women born during 1974–1984.

Educational level	ISCED 0–2	ISCED 3–4	ISCED 5–8
Mean age at first cohabitation	22.306	23.010	24.771
Mean age at first marriage	26.643	27.815	30.058
Mean age at first separation	28.130	28.407	29.765
Mean age at first divorce	35.954	37.078	38.880
Mean age at first re-partnering	32.049	32.576	33.517
Percent ever cohabited	99.861	94.600	94.001
Percent ever married	84.558	74.828	65.927
Percent marriage (cohabitation-marriage)	64.501	61.296	51.726
Percent separation	29.968	28.485	34.017
Percent divorce	30.199	24.949	23.660
Percent re-partnering	74.394	73.810	72.399
Mean age at first birth	27.420	28.126	29.908
Mean age at second birth	29.916	30.683	32.382
Mean age at third birth	31.801	32.542	34.077
Mean age at fourth birth	33.261	34.126	35.481
Completed Cohort Fertility	1.848	1.771	1.671
Fertility gap	0.177	0.254	0.353
Percent 0 children	19.264	22.955	25.738
Percent 1 child	11.078	10.020	11.193
Percent 2 children	43.911	42.245	40.496
Percent 3 children	18.891	18.198	16.776
Percent 4+ children	6.856	6.581	5.798
Miscarriages per live birth	0.171	0.172	0.186
Percent unintended births	19.035	19.413	20.118
Abortion ratio (abortions per 1000 live births)	148.327	152.523	160.896
Percent of births outside coresidential union	2.498	2.121	2.428
Percent IUI births	1.204	1.254	1.462
Percent ART births	2.020	2.238	2.644

ISCED is The International Standard Classification of Education. ISCED 0–2 is a primary level of education, ISCED 3–4 a secondary level of education, and ISCED 5–8 a tertiary level of education.
