# Supplementary material for: Microsimulation reveals that medically assisted reproduction is unlikely to compensate for cohort fertility decline due to increasing maternal ages
Source: Hum Reprod. 2026 Feb 18;41(4):552–62. doi: 10.1093/humrep/deag006 (PMC13061122; doi:10.1093/humrep/deag006)
Supplement: deag006_Supplementary_Table_S5 [file deag006_supplementary_table_s5.pdf]

OXFORD human reproduction

Human Reproduction, 2026, 1–1
https://doi.org/10.1093/humrep/deag006
Supplementary Data

Supplementary Table S5. Completed cohort fertility without medically assisted reproduction by level of education of Dutch women born during 1974–1984.

Educational level	ISCED 0–2	ISCED 3–4	ISCED 5–8
Mean age at first cohabitation	22.288	23.002	24.774
Mean age at first marriage	26.603	27.807	30.052
Mean age at first separation	28.096	28.422	29.801
Mean age at first divorce	35.926	37.070	38.841
Mean age at first re-partnering	32.016	32.557	33.535
Percent ever cohabited	99.859	94.538	93.903
Percent ever married	84.331	74.900	65.892
Percent marriage (cohabitation-marriage)	64.462	61.417	51.706
Percent separation	30.057	28.488	33.998
Percent divorce	30.062	24.845	23.635
Percent re-partnering	74.279	73.672	72.556
Mean age at first birth	27.330	28.060	29.831
Mean age at second birth	29.827	30.602	32.301
Mean age at third birth	31.682	32.449	34.006
Mean age at fourth birth	33.131	33.935	35.373
Completed Cohort Fertility	1.814	1.730	1.624
Fertility gap	0.210	0.294	0.400
Percent 0 children	20.161	24.117	27.253
Percent 1 child	11.306	10.362	11.417
Percent 2 children	43.552	41.622	39.685
Percent 3 children	18.541	17.698	16.219
Percent 4+ children	6.440	6.201	5.425
Miscarriages per live birth	0.168	0.170	0.182
Percent unintended births	19.470	19.875	20.525
Abortion ratio (abortions per 1000 live births)	152.979	156.982	164.672
Percent of births outside coresidential union	2.515	2.161	2.434
Percent IUI births	0.000	0.000	0.000
Percent ART births	0.000	0.000	0.000

ISCED is The International Standard Classification of Education. ISCED 0–2 is a primary level of education, ISCED 3–4 a secondary level of education, and ISCED 5–8 a tertiary level of education.
